# The Landscape of the Tumor Microenvironment in Skin Cutaneous Melanoma Reveals a Prognostic and Immunotherapeutically Relevant Gene Signature

**DOI:** 10.3389/fcell.2021.739594

**Published:** 2021-10-01

**Authors:** Sitong Zhou, Yidan Sun, Tianqi Chen, Jingru Wang, Jia He, Jin Lyu, Yanna Shen, Xiaodong Chen, Ronghua Yang

**Affiliations:** ^1^Department of Dermatology, The First People’s Hospital of Foshan, Foshan, China; ^2^Department of Oncology, First Teaching Hospital of Tianjin University of Traditional Chinese Medicine, Tianjin, China; ^3^Department of Burn Surgery and Skin Regeneration, The First People’s Hospital of Foshan, Foshan, China; ^4^Department of Pathology, The First People’s Hospital of Foshan, Foshan, China; ^5^School of Medical Laboratory, Tianjin Medical University, Tianjin, China

**Keywords:** skin cutaneous melanoma, prognostic biomarker, tumor microenvironment, gene signature, immuno-genomic landscape, clinicopathological characteristics

## Abstract

The tumorigenesis of skin cutaneous melanoma (SKCM) remains unclear. The tumor microenvironment (TME) is well known to play a vital role in the onset and progression of SKCM. However, the dynamic mechanisms of immune regulation are insufficient. We conducted a comprehensive analysis of immune cell infiltration in the TME. Based on the differentially expressed genes (DEGs) in clusters grouped by immune infiltration status, a set of hub genes related to the clinical prognosis of SKCM and tumor immune infiltration was explored.

**Methods:** We analyzed immune cell infiltration in two independent cohorts and assessed the relationship between the internal pattern of immune cell infiltration and SKCM characteristics, including clinicopathological features, potential biological pathways, and gene mutations. Genes related to the infiltration pattern of TME immune cells were determined. Furthermore, the unsupervised clustering method (k-means) was used to divide samples into three different categories according to TME, which were defined as TME cluster-A, -B, and -C. DEGs among three groups of samples were analyzed as signature genes. We further distinguished common DEGs between three groups of samples according to whether differences were significant and divided DEGs into the Signature gene-A group with significant differences and the Signature gene-B group with insignificant differences. The Signature gene-A gene set mainly had exon skipping in SKCM, while the Signature gene-B gene set had no obvious alternative splicing form. Subsequently, we analyzed genetic variations of the two signatures and constructed a competing endogenous RNA (ceRNA) regulatory network. LASSO Cox regression was used to determine the immune infiltration signature and risk score of SKCM. Finally, we obtained 13 hub genes and calculated the risk score based on the coefficient of each gene to explore the impact of the high- and low-risk scores on biologically related functions and prognosis of SKCM patients further. The correlation between the risk score and clinicopathological characteristics of SKCM patients indicated that a low-risk score was associated with TME cluster-A classification (*p* < 0.001) and metastatic SKCM (*p* < 0.001). Thirteen hub genes also showed different prognostic effects in pan-cancer. The results of univariate and multivariate Cox analyses revealed that risk score could be used as an independent risk factor for predicting the prognosis of SKCM patients. The nomogram that integrated clinicopathological characteristics and immune characteristics to predict survival probability was based on multivariate Cox regression. Finally, 13 hub genes that showed different prognostic effects in pan-cancers were obtained. According to immunohistochemistry staining results, *Ube2L6*, *SRPX2*, and *IFIT2* were expressed at higher levels, while *CLEC4E*, *END3*, and *KIR2DL4* were expressed at lower levels in 25 melanoma specimens.

**Conclusion:** We performed a comprehensive assessment of the immune-associated TME. To elucidate the potential development of immune-genomic features in SKCM, we constructed an unprecedented set of immune characteristic genes (*EDN3*, *CLEC4E*, *SRPX2*, *KIR2DL4*, *UBE2L6*, and *IFIT2*) related to the immune landscape of TME. These genes are related to different prognoses and drug responses of SKCM. The immune gene signature constructed can be used as a robust prognostic biomarker of SKCM and a predictor of an immunotherapy effect.

## Introduction

Skin cutaneous melanoma (SKCM) is currently one of the most lethal human malignancies. As the deadliest form of skin cancer, it accounts for almost 75% of skin cancer lethality (Siegel et al., 2021). Although the 5-year survival of early stage SKCM patients exceeds 95% (Thompson et al., 2009), the reported survival time for advanced-stage melanoma barely exceeds 1 year (Fecher et al., 2007). Currently, patients with primary melanoma require surgical resection as a first-line therapy. However, advanced melanoma is highly aggressive, making it insensitive to radiotherapy and chemotherapy (Goodson and Grossman, 2009). The emergence of immune checkpoint inhibitors, such as ipilimumab (Jameson-Lee and Luke, 2021) and nivolumab (Zhao et al., 2020), for melanoma has revolutionized the treatment of SKCM and offers new hope for patients. However, approximately 50% of patients do not benefit from immune checkpoint inhibitors (Hodi et al., 2010; Topalian et al., 2012).

Recently, growing evidence has demonstrated that tumor microenvironment (TME) plays an important role in SKCM progression (Hanahan and Weinberg, 2011; Quezada et al., 2011). TME influences tumorigenesis and metastasis through various biological processes. In contrast to this, TME heterogeneity is also an important cause of alterations in prognosis and sensitivity to immunotherapy in various cancers (Koikawa et al., 2021). Notably, it may regulate the immune response through a variety of mechanisms, thereby affecting the inner metabolism process and immunosuppressive state. Among these, tumor-infiltrating immune cells (TIICs) exhibit tumor-promoting effects according to the tumor type. In most cancers, CD8+ T cells play crucial roles in TME, inhibiting the proliferation and invasion of malignant cells. T cell-mediated immune responses to melanoma antigens have been extensively documented (Marron et al., 2021). Furthermore, immunosuppression may act as an additional tumor burden that fosters tumor growth or immune escape in TME. Hence, a comprehensive understanding of TME is urgently needed to improve the efficacy of immunotherapies.

Considering the positive effect of immunotherapy in SKCM patients, understanding the molecular composition and function of TME is important to facilitate effective diagnosis, prognosis, mitigation, and immunotherapeutic responsiveness of SKCM patients.

We integrated The Cancer Genome Atlas (TCGA)-SKCM-independent cohort and validated the predictive model in four additional independent cohorts (GSE8401, GSE35640, GSE15605, and GSE46517) from Gene Expression Omnibus (GEO) datasets, to develop and verify a new set of personalized immune signature models. We also analyzed clinical and pathological characteristics of all existing SKCM patients, including somatic cell copy number variation (CNV), tumor mutation burden (TMB), and gene variable splicing. Several bioinformatics methods were employed to estimate the abundance of immune cell infiltration in SKCM patients, and the correlation between genomic characteristics of the immune landscape and pathological characteristics and prognosis of SKCM was determined. Therefore, in the present study, we sought to develop and evaluate the risk-based model of six novel immune-related genes (*EDN3*, *CLEC4E*, *SRPX2*, *KIR2DL4*, *Ube2L6*, and *IFIT2*). In addition, immunohistochemistry (IHC) staining and qRT-PCR were used to verify the difference in the expression level of the novel 6-gene signature in 25 frozen SKCM tissue samples and normal tissues. Notably, gene signatures that affect the immune landscape may be closely related to different prognosis and treatment responses of SKCM. Accordingly, we established an unprecedented set of immune signatures that can be used as robust prognostic biomarkers and predictors of the immunotherapy effects in SKCM patients.

## Materials and Methods

### Acquisition and Preprocessing of the SKCM Expression Datasets

Data from two publicly available datasets were included in the study. RNA-seq data were extracted for 471 patients, in addition to clinical features, from TCGA-SKCM cohort (Tomczak et al., 2015). Clinical data were downloaded from the University of California Santa Cruz Xena browser^1^. Somatic CNV data and TMB were downloaded from the Genomic Data Commons Data Portal (Grossman et al., 2016). Data on alternative splicing events were downloaded from TCGASpliceSeq database. “RCircos” (Zhang et al., 2013) package was used to generate a map for the genome-wide CNV analysis of SKCM patients using 23 pairs of chromosomes. Somatic mutation data were downloaded in mutation annotation format, and the maftools package (Mayakonda et al., 2018) was used for visualization. We obtained SKCM microarray data from two GeneChips (GPL570 [HG-U133_Plus_2] Affymetrix Human Genome U133 Plus 2.0 Array and GPL96-57554 [HG-U133A] Affymetrix Human Genome U133A Array) in GSE84014 (Xu et al., 2008), GSE356405 (Dreno et al., 2018), GSE156056 (Raskin et al., 2013), and GSE46517 (He et al., 2020) from GEO^1^ as validation sets. The limma package (Ritchie et al., 2015) in R was used for gene expression normalization, while the sva (Leek et al., 2012) package (3.20) was used to correct for plate batch effects.

### TIICs Analysis and Clustering of Samples

CIBERORT (Newman et al., 2015), an algorithm that quantifies the proportion of TIICs with 547 signature genes to infer the representation in bulk tumor transcriptomes, was used to evaluate the cell components of TIICs. The proportions of 22 types of TIICs in the cohort from TCGA were estimated. Cluster analysis was performed using an unsupervised hierarchical clustering method, an algorithmic approach that groups individuals with similar observations based on the Euclidean distance method. Three immune microenvironment subtypes were defined using the ConsensusClusterPlus package (Wilkerson and Hayes, 2010). The procedure was repeated 1,000 times to stabilize the stratification.

### Differential Gene Expression Pattern Clustering Analysis

To determine genes associated with TME cell infiltration patterns, we divided patients into three different pattern types according to TME; these types were defined as TME cluster-A, -B, and -C. We further analyzed common differentially expressed genes (DEGs) between the three groups of samples and, based on the significance of the differential expression changes of the specific genes, divided the samples into two groups of signature genes: A and B. Signature gene-A was an immune-related specific gene with a significant difference, and Signature gene-B was an insignificantly different part of an immune-related specific gene. The limma package was used to analyze DEGs between these three groups of SKCM patients, and the significant DEGs were defined as genes with an absolute log value of fold-change >1 and FDR <0.05. The overlapping DEGs among the three groups were further analyzed as specific genes using the VennDiagram (Venn diagram) package (Chen and Boutros, 2011). The k-means clustering algorithm, which is an unsupervised clustering method, was used to cluster these specific genes into meaningful groups in the GEO datasets according to the expression of specific genes in TME cluster-A, -B, and -C. Meanwhile, based on changes in gene expression, specific genes were divided into two groups: Signature gene-A and -B. Among them, Signature gene-A and Signature gene-B were obtained by cluster analysis of the three subtypes, where Signature gene-A was highly expressed in cluster A and relatively less expressed in clusters B and C.

### RNA Sequence Expression Analysis in Gene Expression Profiling Interactive Analysis

Gene Expression Profiling Interactive Analysis (GEPIA)^2^ is an online data processing webpage containing RNA sequence expression information of 9,736 tumors and 8,587 normal tissue samples (Tang et al., 2019). The differential expression of hub genes between tumor and normal tissues, correlations, and survival prediction was determined using the GEPIA database. Student’s *t*-test was used to analyze the correlation between the expression and clinicopathological features. Statistical significance was set at *p* < 0.05.

### Gene Set Enrichment Analysis

Gene Ontology (GO) analysis was performed to illustrate the unique biological significance of gene expression of signature genes. Gene functions were categorized into three series: cellular components, molecular functions, and biological processes (BPs). Crucial pathways were identified using Kyoto Encyclopedia of Genes and Genomes pathway analysis. GO annotations were visualized using the R package clusterProfiler (Yu et al., 2012). Gene Set Enrichment Analysis (GSEA; Subramanian et al., 2005) is a calculation method for identifying the potential biological mechanisms between two biological states. GSEA was conducted in the Molecular Signatures Database (MSigDB)^3^, which provided hallmark gene sets to predict BPs between normal and SKCM samples.

### Evaluation of Patient Biological Characteristics

We further analyzed correlations between different groups and some biologically related processes. Gene sets for storing genes related to certain BPs, including immune checkpoints, antigen processing, CD8+ T cells, and epithelial–mesenchymal transition (EMT) markers, such as EMT1, EMT2, and EMT3, angiogenesis characteristics, pan-fibroblast TGF-β response characteristics, WNT characteristics, DNA damage repair, mismatch repair, nucleotide excision repair, DNA replication, and antigen processing and presentation, were collected.

### Establishment of the Immune Characteristic Model and Clinical Prediction Model

We adopted a two-step method to establish a signature-based risk score. First, univariate Cox regression was used to analyze the impact of signature genes on the prognosis of SKCM patients according to the cutoff, with *p* < 0.05. The most commonly used model to establish clinical prognosis and survival-related models is the Cox proportional hazards model owing to its flexibility and relative robustness. The Cox grade-scoring test was used for repeated prediction and prognostic analyses. The Cox grade-scoring test was robust to outliers. Accordingly, outliers were removed for the sensitivity analysis. The potential prognostic hub genes were validated to enhance risk profiling after surgery and to define new targets in the prediction models for SKCM patients. To prove the significance of risk score combined with clinicopathological characteristics for a personalized evaluation of patient prognosis, we first tested the expression correlation of hub genes in different tumors in TCGA database and the predictive ability of the risk score for the prognosis of patients with different tumors. Multiple regression was the main analysis method for building immune prediction models. Multiple regressions were performed using standard statistical methods. In addition, we fitted the multivariate Cox regression model using the least absolute contraction and selection operator (LASSO). LASSO regression is a regularized approach commonly used for high-dimensional predictor selection. A system of risk score was established using the LASSO Cox proportional hazards model to identify gene signatures for predicting the overall survival (OS) of SKCM. A predictive score was developed using the weighted sum of genes, with the coefficients of the LASSO regularization.


riskScore=∑Coefficient(hubgenei)*mRNAExpression(hubgenei)

Factors related to OS and clinical pathological characteristics were evaluated using univariate and multivariate analyses with Cox and logistic regression, respectively. Variables with a value of *p* < 0.05 in the multivariate analysis were included in the prognostic model. The performance and discriminative ability were assessed using Harrell’s concordance index. Nomograms were constructed to predict the 3-year, 5-year, and 10-year survival rates of SKCM patients based on predictive models with identified prognostic factors. Calibration is defined as a prediction from the nomogram compared with the observed outcomes.

### IHC Validation

Tumor tissues were collected from 25 melanoma patients in the Han Chinese group from 2015 to 2020. Informed consent was obtained from patients, and the study was approved by the First People’s Hospital of the Foshan Subject Review Board. Paraffin-embedded tissues were sectioned to be 4 μm thick for IHC analysis. Antigen retrieval was performed by incubating the samples in citrate buffer (pH 6.0) for 15 min at 100°C in a microwave oven. After blocking with a mixture of methanol and 0.75% hydrogen peroxide, sections were incubated overnight with appropriate dilutions of primary antibodies (CLEC4E, 1:500, Sigma; END3, 1:500, Sangon Biotech; IFIT2, 1:500, Proteintech; SRPX2, 1:600, Proteintech; Ube2L6, 1:500, Abcam; KIR2DL4, 1:500, Abcam), followed by incubation with a secondary antibody conjugated with HRP (goat anti-rabbit, 1:500, Cell Signaling Technology). The sections were washed three times with phosphate buffer saline and incubated with AEC (ZSGB-BIO). All specimens were examined by the cross-product (H score) of the percentage of tumor cell staining at each of three staining intensities. The intensity of immunopositivity was scored as follows: none, 0; weak, 1; moderate, 2; and strong, 3. For example, a particular tumor may have 50% cell staining at an intensity of 1 and 50% of cell staining at an intensity of 3, for a combined H score of 200 [(50 × 1) + (50 × 3) = 200], which yields a range from 0 to 300. The final score was graded by the H score as follows: low, H score 0–100; moderate, H score 101–200; and high, H score 201–300.

### Statistical Analysis

All statistical analyses were performed using R version 3.6.2. Differences in continuous variables between the two groups were estimated by independent Student’s *t*-test, and the differences between non-normally distributed variables were analyzed using the Mann–Whitney U rank-sum test. If the normality test failed, Kruskal–Wallis One Way ANOVA on Ranks was performed. Pearson’s χ^2^ test or Fisher’s exact test was used to compare qualitative variables. *p*-Values and hazard ratios were obtained from univariate Cox proportional hazards regression models using the R package, *survival* (Zeng et al., 2019). The log-rank test was used to evaluate the significance of the difference in survival time between the two groups. Receiver operating characteristic curve analysis was conducted using the pROC package (Robin et al., 2011) to evaluate the prognostic capabilities of different risk models and the time-dependent AUC values. Univariate and multivariate Cox analyses were used to determine independent prognostic factors. All statistical tests were two-sided, and statistical significance was set at *p* < 0.05.

## Results

### Immune Infiltration Analysis Related to SKCM Patients

**FIGURE 1:**
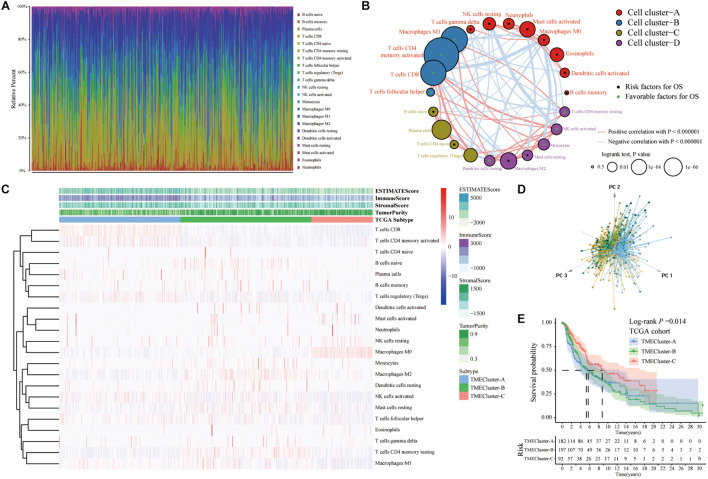
Immune cellprotect infiltration in TCGA-SKCM database. **(A)** Histogram showing the infiltration of 22 different specific immune cells in each sample CIBERSORT. Different colors indicate different tumor-infiltrating immune cells. **(B)** The interaction between immune cells in TME in TCGA-SKCM. Cell cluster-A, red; Cell cluster-B, blue; Cell cluster-C, brown; Cell cluster-D, purple. The size of the circle represents the impact of each TME cell type on survival, and the log-rank test was used for analysis. The green part in the center of the circle indicates that the cell protects against overall survival, and the black indicates the risk to overall survival. The lines connecting TME cells represent cell interactions. The thickness of the line represents the correlation strength estimated by Spearman correlation analysis, in which positive correlation is represented by red and negative correlation is represented by blue. **(C)** Heatmap showing the infiltration of TIICs in 471 SKCM patients in TCGA database combined with the immune score. Unsupervised clustering grouping the samples into three major clusters. **(D)** The PCA of the gene expression profile distinguishes patients in the TME cluster-A, -B, and -C groups in TCGA-SKCM. The results can distinguish three different immune infiltration pattern samples (TME cluster-A: blue, TME cluster-B: yellow, TME cluster-C: green). **(E)** The Kaplan–Meier curve of the patient’s overall survival (OS) shows that TME infiltration is significantly related to the overall survival (Log-rank test, *p* = 0.014).

We obtained the gene expression data of SKCM patients from TCGA database(Table 1) and analyzed 22 different immune cell infiltrations in each sample using the CIBERSORT algorithm (Newman et al., 2015; Figure 1A). The TME cell network depicted interactions between tumor immune cells (Supplementary Figure 1). The comprehensive status of the cell lineage and its impact on the overall survival of patients with SKCM were also analyzed (Figure 1B). The TME cell network demonstrated that cell clusters B, C, and D were positively correlated with each other, including CD8+ T cells, naïve B cells, plasma cells, CD4 naïve T cells, activated NK cells, monocytes, resting dendritic cells, regulatory T cells, monocytes, and CD4 memory activated T cells. Moreover, there was a significant positive correlation among immune cells in cluster A, such as between gamma delta T cells, resting NK cells, neutrophils, activated mast cells, M0 macrophages, eosinophils, and activated dendritic cells. Meanwhile, M0 macrophages, activated CD4 memory T cells, and monocytes showed a significant negative correlation. NK cell resting and NK cell activation showed the same significant negative correlation. An exploratory analysis was also performed to measure survival benefits and potential risks. Results showed that CD4+ T cells, CD8+ T cells, activated NK cells, regulatory T cells, dendritic cells, and M1 macrophages were associated with shortened OS, while resting NK cells, neutrophils, M0 macrophages, and dendritic cells were activated and associated with prolonged OS. To construct the best clusters and classification, we used the ConsensusClusterPlus package to assess the stability of the clustering structure and divided SKCM patients into TME cluster-A, -B, and -C. Unsupervised hierarchical clustering was used to analyze the normalized immune cell fractions. The heatmap showed correlation between the infiltration abundance of 22 types of immune cells and immune scores in the three groups (Figure 1C). Results showed that patients with TME cluster-A had higher immune scores, whereas patients with TME cluster-B and -C mainly had tumor purity and stromal scores. In addition, the principal component analysis results showed that based on the expression data of SKCM patients, the three TME cluster groups could be significantly distinguished (Figure 1D). Survival analysis related to the TME phenotype showed that TME cluster-C (*N* = 92) was associated with a better prognosis (log-rank test, *p* < 0.001) (Figure 1E).

**TABLE 1:** Baseline data of SKCM patients in TCGA database.

Variables	All (*n* = 422)	Low risk (*n* = 205)	High risk (*n* = 217)	*p*-value
Gender				0.02*
—Male	264(62.6%)	117(57.1%)	147(67.7%)	
—Female	158(37.4%)	88(42.9%)	70(32.3%)	
Age				0.68
—<60	220(52.1%)	109(53.2%)	111(51.2%)	
—≥60	202(47.9%)	96(46.8%)	106(48.8%)	
T stage				< 0.001***
—T1 & T2	147(34.8%)	91(44.4%)	56(25.8%)	
—T3 & T4	236(55.9%)	93(45.4%)	143(65.9%)	
N stage				0.85
—N0	218(51.7%)	108(52.7%)	110(50.7%)	
—N1	72(17.1%)	37(18.0%)	35(16.1%)	
—N2	47(11.1%)	24(11.7%)	23(10.6%)	
—N3	55(13.0%)	32(15.6%)	23(10.6%)	
M stage				0.836
—M0	392(92.9%)	192(93.7%)	200(92.2%)	
—M1	23(5.5%)	10(4.9%)	13(6.0%)	
Pathologic stage				0.03*
—I	83(19.7%)	54(26.3%)	29(13.4%)	
—II	146(34.6%)	58(28.3%)	88(40.6%)	
—III	170(40.3%)	83(40.5%)	87(40.1%)	
—IV	23(5.5%)	10(4.9%)	13(6.0%)	
Type				< 0.001***
—Primary	97(23.0%)	28(13.7%)	69(31.8%)	
—Metastatic	325(77.0%)	177(86.3%)	148(68.2%)	
Status				< 0.001***
—Alive	232(55.0%)	139(67.8%)	93(42.9%)	
—Dead	190(45.0%)	66(32.2%)	124(57.1%)	

*p < 0.05; ***p < 0.001; ns, no significance.

**FIGURE 2:**
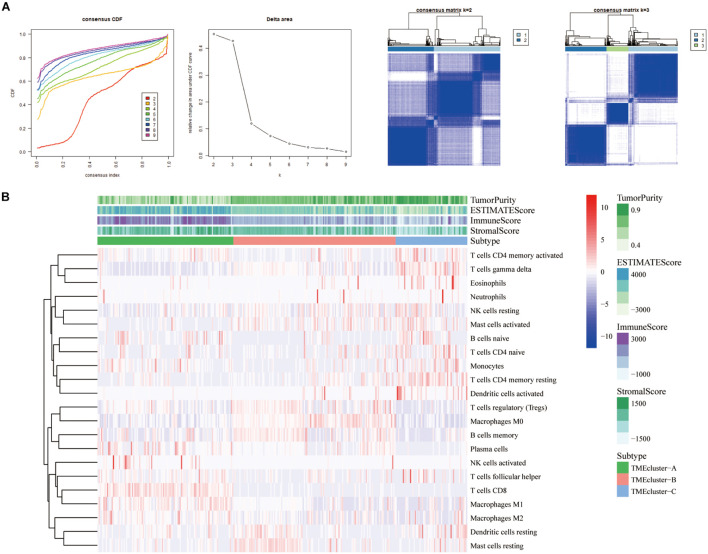
Three types of SKCM patients in the GEO database based on TIICs infiltration. **(A)** Hierarchical clustering determined the number of clusters of the immune cell infiltration patterns of 310 SKCM patients in the GEO database (GSE8401, GSE35640, GSE15605, and GSE46517) and repeats 1,000 times to ensure the stability of the classification. **(B)** Heatmap displaying the expression of tumor-infiltrating immune cells in SKCM patients from GEO database was reviewed.

We selected and analyzed the gene expression profile data and immune cell infiltration abundance of SKCM samples from the GEO database. The ConsensusClusterPlus package was used to evaluate the stability of the clustering (Figure 2A). Subsequently, the heatmap showed that patients with TME cluster-A had higher immune scores (Figure 2B), which was consistent with the results in TCGA database. This finding also aligned with that of previous studies that have reported melanoma being a highly immune-dependent malignant tumor.

### Construction of the TME-Related Signature Gene in SKCM Patients

**FIGURE 3:**
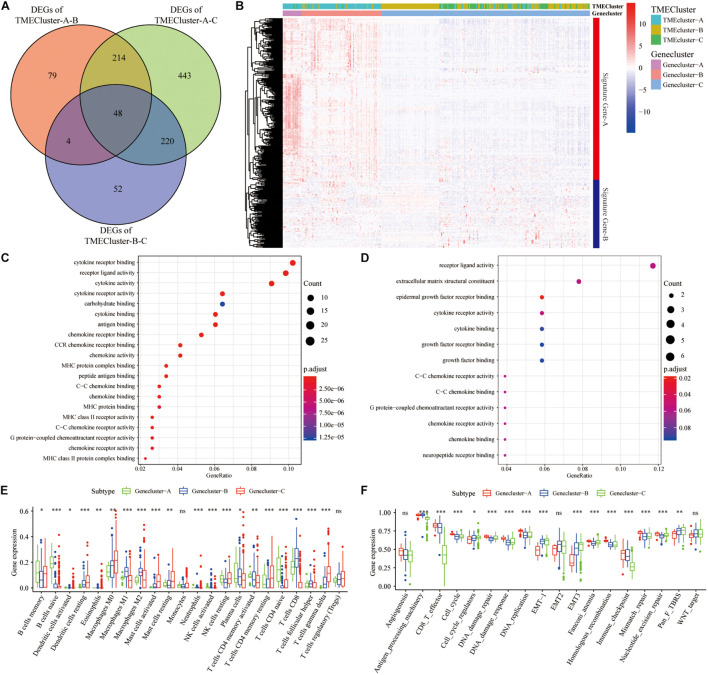
Construction and functional annotation of TME-related characteristic genes. **(A)** The Venn diagram showing the DEGs between the three TME clusters in TCGA database. **(B)** Based on the gene expression profile of SKCM patients in the GEO database, unsupervised analysis and hierarchical clustering of DEGs were carried out, divided into GeneCluster-A, -B, and -C. According to their expression changes, DEGs were classified into Signature gene-A and -B. **(C,D)** Gene Ontology (GO) enrichment analysis of Signature gene-A **(C)** and -B **(D)** gene sets. **(E)** The expression of cells in the three GeneCluster groups. **(F)** The enrichment of different pathway characteristics (immune-related features, mismatch-related features, and matrix-related features) in three different GeneCluster groups (^∗^*p* < 0.05, ^∗∗^*p* < 0.01, ^∗∗∗^*p* < 0.001).

TME has a crucial impact on tumor epigenetics, metastasis, and immune escape. We used the limma package to analyze the 486 DEGs among the three groups of TME cluster in TCGA database to determine the potential biological characteristics of different TME phenotypes. Similarly, 330 DEGs were analyzed in the GEO datasets (Figure 3A). Unsupervised clustering using these DEGs provided three distinct clusters: TME cluster-A, -B, and -C (Figure 3B). Simultaneously, according to the differential expression of DEGs in different clusters, genes were further divided into Signature gene-A and -B. GO enrichment analysis showed that Signature gene-A and -B had different unique BPs. Signature gene-A was associated with the overexpression of immune-activated genes (Figure 3C), while Signature gene-B showed upregulation of genes related to stromal and transmembrane receptors (Figure 3D). There were significant differences in the infiltration of TME cells (Figure 3E) and the enrichment of related biological pathways (Figure 3F) between the three GeneClusters, which was consistent with our functional enrichment results.

### CeRNA Regulatory Network Construction and Signature Gene Expression in SKAM Patients

**FIGURE 4:**
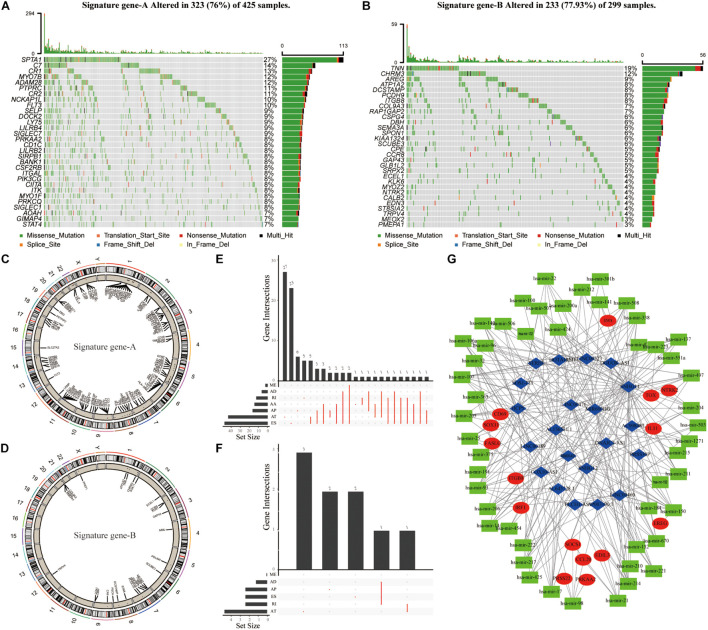
The genetic variation and ceRNA network of Signature gene-A and -B in SKCM patients. **(A,B)** The mutation maps of Signature gene-A and -B, respectively, in SKCM patients. The mutation information of each gene was displayed in the waterfall chart, and various colors indicated different mutation types; the upper section of the legend showed the mutation load. **(C,D)** Signature gene-A and -B are, respectively, on 23 chromosomes of SKCM patients. The CNV difference and its position on the above. **(E,F)** UpSet diagram showing the variable splicing of Signature gene-A and -B in SKCM patients. **(G)** Based on the expression of Signature gene-A and -B in SKCM patients. To construct a ceRNA regulatory network of mRNA–miRNA–LncRNA and summarize the complex relationship between Signature gene-A and -B (red dots), immune-related LncRNA (blue dots), and miRNA targeting LncRNA (green dots).

To further analyze differences between Signature gene-A and -B gene sets and determine whether these differences have a profound impact on cancer genetics, we analyzed genetic levels of single nucleotide polymorphisms. CNV and SNP analyses showed that there were obvious mutations in both gene sets. Notably, somatic SPTA1 mutations were more frequent in TME cluster-A (Figure 4A). TNN was the most frequently observed somatic mutation in TME cluster-B (Figure 4B). Moreover, the examination of this frequency change in CNV revealed that CNV changes were common in both groups and most were concentrated on the amplification of copy numbers. We then elucidated positions of these CNV changes on the chromosome (Figures 4C,D). In the AS analysis, Signature gene-A mainly had exon skipping (ES) in SKCM patients (Figure 4E), while Signature gene-B had no obvious AS forms (Figure 4F). In addition, Signature gene-A and -B gene sets may have a certain significance for the prognosis of SKCM. Further research is necessary for the interaction between immune-related molecules. Based on the gene expression of Signature gene-A and -B, we screened out lncRNAs that may be related to immunity using correlation coefficients >0.4 and *p* < 0.05. The starBase database^4^ was used to obtain targeted differentially expressed miRNAs to construct the regulatory ceRNA network of the mRNA–miRNA–LncRNA interaction (Figure 4G).

### Construction of the Immune-Related Prognostic Gene Signature

**FIGURE 5:**
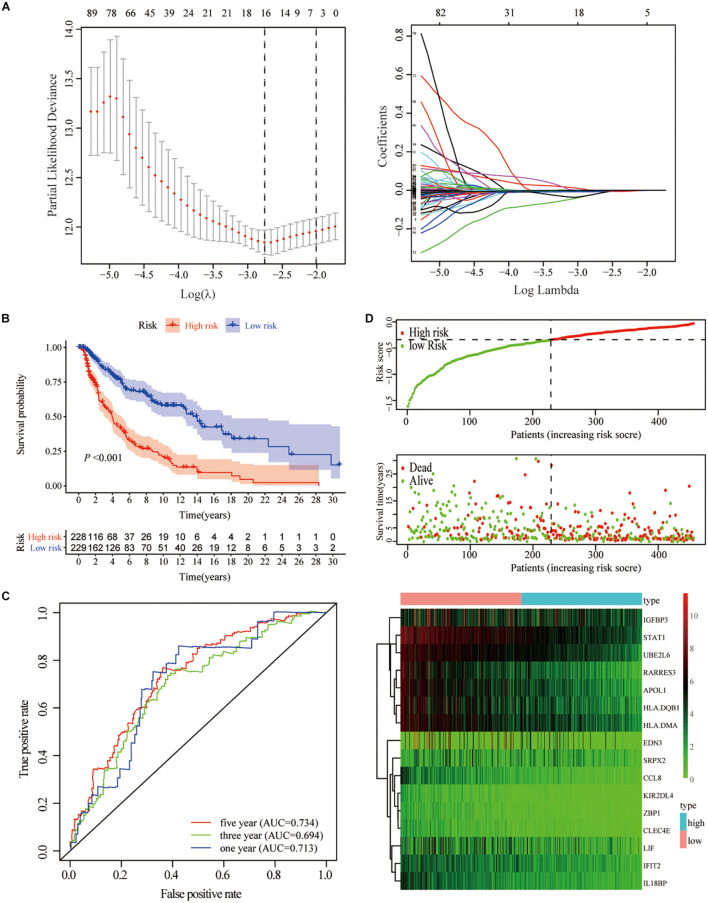
Construction of gene-based risk prediction model. **(A)** LASSO Cox regression analysis identified 13 hub genes closely related to OS of TCGA-SKCM. **(B)** Kaplan–Meier plots assessed the impact of risk scores on the OS of SKCM patients in TCGA. **(C)** Time-dependent ROC curve analysis of the risk score. **(D)** The distribution of risk scores, the expression values of the 13 hub genes, and the survival status of SKCM patients ranked according to the risk scores.

To predict the impact of immune characteristics on the prognosis of patients better, we constructed a new prognostic-related risk scoring system. The Signature gene-A and-B were incorporated into univariate Cox analyses, and 233 genes related to prognosis were obtained (*p* < 0.05). The LASSO Cox analyses were further used for dimensionality reduction and model construction, and finally, a total of 16 hub genes were included in the risk scoring model (Figure 5A). A risk score (RS) formula was established by including individual normalized gene expression values weighted by their LASSO Cox coefficients. The risk score for each SKCM patient was calculated according to the locking of the coefficients in each gene signature. The high- and low-risk groups were divided according to the median risk score. Kaplan–Meier analysis showed that patients with high-risk scores had a relatively poor prognosis (Figure 5B). The time-dependent receiver operating characteristic curve analysis also showed that the risk score had a good predictive ability for OS (Figure 5C). The area under the curve (AUC) of 1-year, 3-year, and 5-year OS was 0.713, 0.694, and 0.734, respectively. The distribution of each patient’s risk score, survival status, and gene expression map is shown in Figure 5D.

### GSEA

**FIGURE 6:**
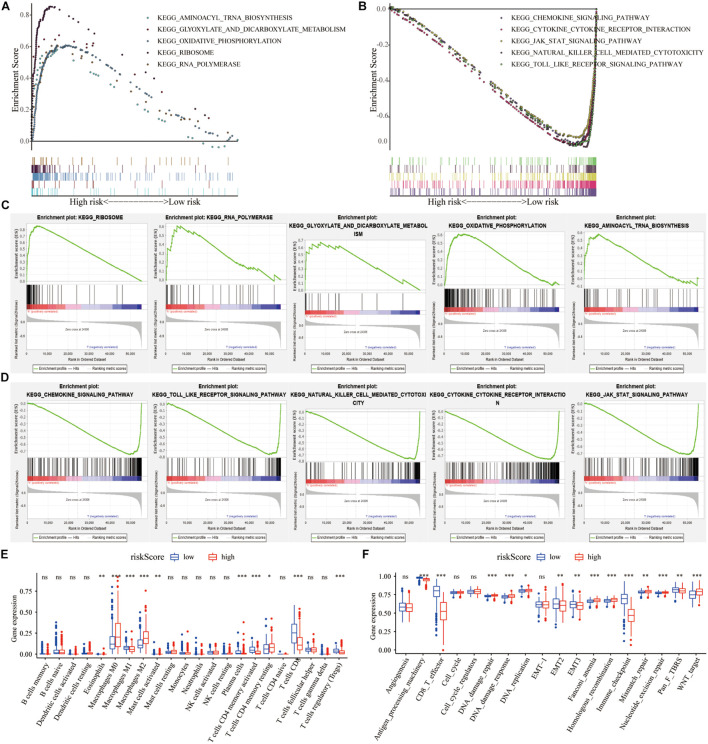
The regulation of risk score on tumor gene expression and biological processes. **(A,B)** GSEA revealed the most significant hallmarks with high- and low-risk clusters. **(C,D)** Enrichment of representative pathways between high- and low-risk patients in GSEA analysis. **(E)** TIICs in the high- and low-risk groups. **(F)** The enrichment of different pathway characteristics (immune-related characteristics, mismatch-related characteristics, and matrix-related characteristics) in high- and low-risk patients (^∗^*p* < 0.05, ^∗∗^*p* < 0.01, ^∗∗∗^*p* < 0.001).

We analyzed the impact of the high- and low-risk groups on the biologically relevant functions of SKCM patients. GSEA revealed that pathways related to metabolism and oxidative phosphorylation were mainly enriched in the high-risk group (Figures 6A,C), while pathways related to immune response, including cytokine signaling pathway, JAK–STAT signaling pathway, and natural killer cell-mediated cytotoxicity, were significantly enriched in low-risk patients (Figures 6B,D). At the same time, expression levels of TME cells (Figure 6E), as well as some other pathways, such as angiogenesis, mismatch-related features, and stromal-related features (Figure 6F), were significantly different between the high- and low-risk groups of SKCM patients (*p* < 0.05).

### Correlation Analysis of the Risk Score and Clinicopathological Characteristics

**FIGURE 7:**
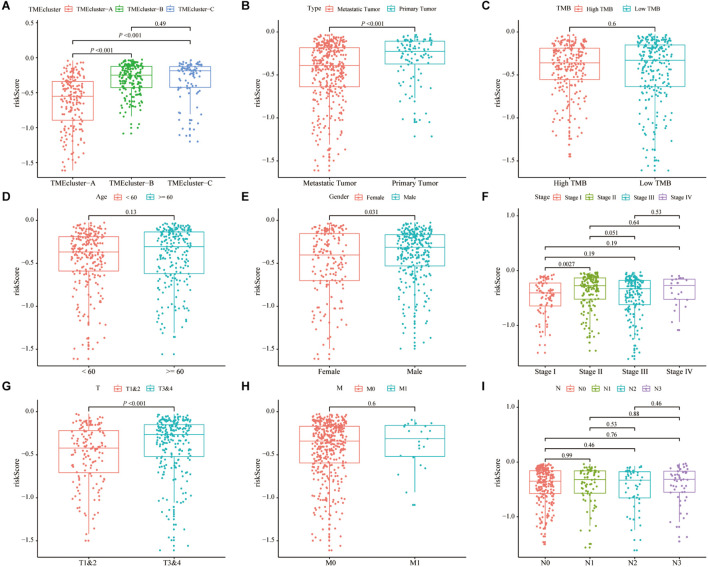
Correlation analysis of risk score and clinicopathological characteristics of SKCM patients in TCGA. **(A–C)** Correlation analysis between risk score and different patient types showed that risk score was related to TME cluster grouping (*p* < 0.001) **(A)** and tumor type (*p* < 0.001) **(B)**. There was no significant correlation with the patient’s tumor mutation burden (TMB) (*p* = 0.6). **(D–I)** The correlation analysis between the risk score and different clinicopathological characteristics of SKCM patients showed that the risk score and gender (*p* = 0.01) **(D)**, stage (*p* = 0.0027) **(E)**, and T stage (*p* < 0.001) **(F)** are related, but there is no significant correlation between the patient’s age **(G)**, M stage **(H)**, and N stage **(I)**.

We assessed the correlation between the risk score and the clinicopathological characteristics of SKCM. The analysis results showed that the low-risk score was correlated with TME cluster-A classification (*p* < 0.001; Figure 7A) and metastatic SKCM (*p* < 0.001; Figure 7B). In addition, the low-risk score was correlated with gender (*p* = 0.031; Figure 7E), and significantly correlated with pathological stage (*p* = 0.0027; Figure 7F) and T stage (*p* < 0.001; Figure 7G). There was no significant correlation between the risk score and TMB, age, M stage, and N stage (*p* > 0.05; Figures 7C,D,H,I and Table 2).

**TABLE 2:** Univariate and multivariate Cox analyses of patients’ OS prediction based on risk score in TCGA database.

Variables	Univariate Cox analysis		Multivariate Cox analysis	
	HR (95% CI)	*p*-Value	HR (95% CI)	*p*-value
Age (≥ 60 vs.<60)	1.47(1.09−1.98)	0.009**	1.44(1.07−1.94)	0.02*
Gender (male vs. female)	1.04(0.77−1.42)	0.78	0.97(0.71−1.32)	0.84
Stage (III + IV vs. I + II)	1.76(1.31−2.37)	< 0.001***	1.96(1.45−2.64)	< 0.001***
Type (Metastatic vs. primary)	0.36(0.22−0.59)	< 0.001***	0.47(0.29−0.76)	0.002**
riskScore (high vs. low)	11.49(6.10−21.64)	< 0.001***	11.10(5.89−20.92)	< 0.001***

*p < 0.05; **p < 0.01; ***p < 0.001; ns, no significance.

### Evaluation and Validation of the Prognostic Signature

**FIGURE 8:**
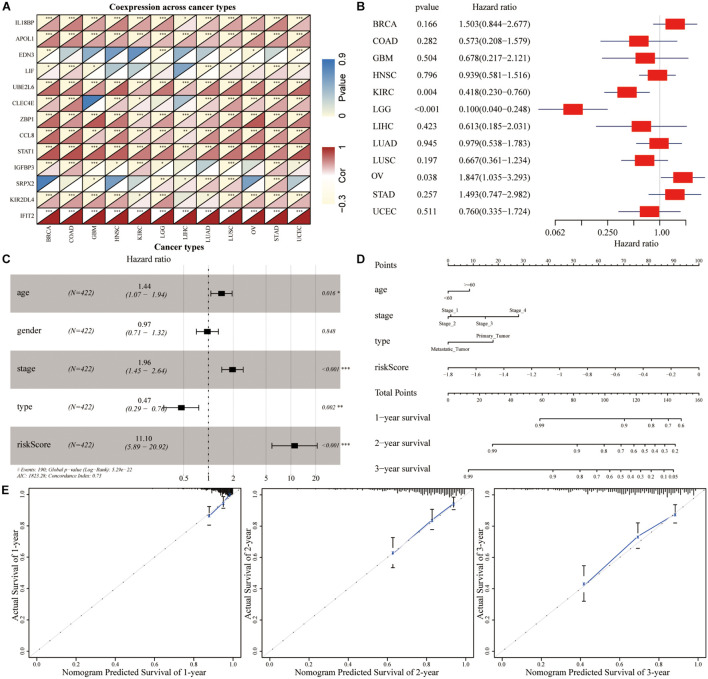
The predictive power of genetic risk score on the prognosis of pan-cancer. **(A)** Correlation analysis between the expressions of 13 hub genes in pan-cancer. **(B)** Subgroup analysis of the prognostic value of the risk score in pan-cancer in TCGA dataset. **(C)** Multivariate Cox regression analysis of risk score combined with clinicopathological characteristics. **(D)** Nomogram prediction of the 1-, 2-, and 3-year survival probability. **(E)** The calibration curve of the nomogram showed that the prediction model had a good predictive value for the prognosis of SKCM patients at 1, 2, and 3 years.

Correlation analysis revealed that the expression of 13 hub genes in different tumors was significantly correlated (Figure 8A). The heterogeneity between tumors caused the risk score to have different prognostic effects on different cancers (Figure 8B). Univariate and multivariate Cox analyses showed that the risk score was an independent risk factor for predicting the prognosis of patients with SKCM (Figure 8C). We included the index of *p* < 0.05 in the multivariate Cox model to construct a nomogram to predict the 1-, 2-, and 3-year survival probability of SKCM patients. The C-indexes [0.732 (95% CI: 0.697–0.767)] of the nomogram were used to calculate the discriminative ability of the nomogram, showing a high degree of discrimination (Figure 8D). The calibration also showed a great agreement between the 1-year, 2-year, and 3-year OS estimates and the actual observed values of SKCM patients through a comparison to the nomogram (Figure 8E).

### Prognostic Value of Hub Genes in SKCM Patients

We focused on the potential prognostic value of EDN3, CLEC4E, SRPX2, KIR2DL4, UBE2L6, and IFIT2 as immune scores for melanoma patients. In the group of novel hub genes, low expression levels of *UBE2L6*, *KIR2DL4*, *IFIT2*, and *CLEC4E* were observed in tumor cells, while high expression levels of *SRPX2* and *EDN3* were found in SKCM. However, only *UBE2L6* and *IFIT2* showed significant expression differences in TCGA-SKCM cohort. A larger sample size may be required to verify the differential expression of these molecules in SKCM. The open online tool, GEPIA, was used to analyze the prognostic value of these novel hub genes. We found that the low expressions of *UBE2L6*, *KIR2DL4*, *IFIT2*, and *CLEC4E* were significantly associated with poor prognosis in SKCM.

### Preliminary IHC Validation in Melanoma Specimens

**FIGURE 9:**
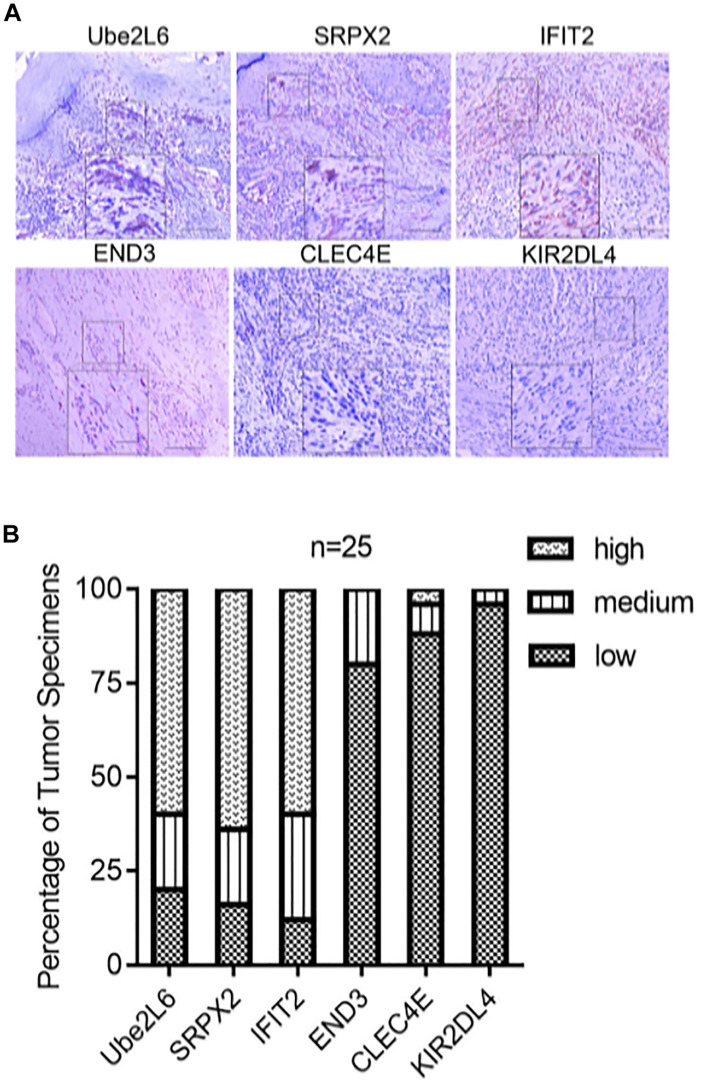
**(A)** Pictures of inmunohistochemical staining of Ube2L6, SRPX2, IFIT2, END3, CLEC4E, and KIR2DL4. **(B)** Higher expressions of Ube2L6, SRPX2, and IFIT2 were presented, while lower expressions of CLEC4E, END3, and KIR2DL4 were shown in melanoma tissues (*n* = 25) by IHC staining.

We evaluated the expression of six novel genes in melanoma tissues. The IHC staining results showed that *Ube2L6*, *SRPX2*, and *IFIT2* were expressed at higher levels, while *CLEC4E*, *END3*, and *KIR2DL4* were expressed at lower levels in 25 melanoma specimens (Figure 9).

## Discussion

The incidence of SKCM continues to increase annually. The recurrence and metastasis of SKCM caused by functional variations in TME markedly contribute to the extremely poor prognosis of SKCM. TME plays a key role in the response to immunotherapy. Several treatments have improved the survival rate of patients with advanced diseases, such as radiotherapy, chemotherapy, and immunotherapy (Wagner et al., 2019). In particular, the application of immunotherapy in SKCM has enabled remarkable breakthroughs (Mitchell et al., 2018). However, there are still many patients who exhibit resistance to cancer immunotherapy and adoptive cell therapy due to the immunosuppressive barriers that exist in TME (Hu et al., 2020). Therefore, there is an urgent need to determine more therapeutic targets and prognostic biomarkers based on TME. In this study, we conducted a comprehensive assessment of immune cell infiltration in TME, identified novel tumor immune subtypes, and assessed the prognostic value of immune cells in SKCM patients. The prognostic characteristics proposed herein are reliable for predicting the survival of patients with SKCM.

To verify the key role of immune cell-infiltrated TME in SKCM, we performed independent microarray data analysis on four datasets in the GEO database. Notably, cross-platform research increased the reliability of our novel prognosis model. We used the CIBERSORT algorithm to evaluate the immune cell infiltration status of the SKCM cohort transcriptome data in TGCA database comprehensively and obtained 22 different immune cell infiltration abundances in each patient. We depicted the tumor immune cell interaction network, including the overall situation of the cell lineage and its impact on the overall survival of SKCM patients, which was reviewed and verified in the SKCM patient cohort from the GEO database. To evaluate the tumor heterogeneity and interaction with TME that can guide better and earlier targeted treatments better, we hierarchically clustered tumor samples based on essential differences in immune cell infiltration patterns. Principal component analysis (PCA) based on the expression profile data of SKCM patients could distinguish the three TME cluster groups well. Thus, we proceeded to analyze the immune-related scores of three groups, tumor purity, and matrix scores. Patients with TME cluster-A had higher immune scores, whereas patients with TME cluster-B and -C mainly had tumor purity and matrix scores. In the GEO database, the GSE8401, GSE35640, GSE15605, and GSE46517 datasets verified the gene expression profile data and immune cell infiltration of SKCM patients and revealed consistent clustering results. To gain a new understanding of the relationship between the above grouping and TME phenotype, we conducted a series of survival analyses and found that TME cluster-C (*n* = 92) was associated with a better prognosis among the three clusters.

We explored the potential biological characteristics of the different TME phenotypes and performed a different analysis on the three TME clusters and displayed the intersection of 486 DEGs using a Venn diagram. Based on the above differential genes, we finally obtained 330 repeated DEGs from the four datasets in the GEO database. We used unsupervised clustering to divide SKCM patients into three different subtypes based on the expression of DEGs, namely GeneCluster-A, -B, and -C. According to the expression of DEGs in the different groups, they were divided into Signature gene-A and -B. We confirmed that the expression of these genes was quite different between the two clusters due to various tumor heterogeneities. These clusters were also clearly associated with different mutational patterns. GO enrichment analysis showed that Signature genes-A and -B had different unique BPs. Immune-activating genes are associated with the activation of immune surveillance and immune activation during tumor immunization; Signature gene-A involves the overexpression of immune-activated genes, and the gene set overexpressing Signature gene-B is mainly demonstrated by upregulation of genes related to the matrix and transmembrane receptors. Such finding suggests that the Signature gene-A gene set may have an impact on the immunophenotypic landscape of TME. In addition, the analysis results showed that there were significant differences in the expression of TME cells and the enrichment of some related biological pathways among the three GeneCluster groups.

To explore causes of tumor immune microenvironment heterogeneity more deeply, we analyzed the tumor mutation-related characteristics of patients. The CNV information of SKCM was downloaded in addition to the somatic mutation data and TMB of each patient in Signature gene-A and -B. We also explored SNPs and CNVs in SKCM patients. The gene variable splicing information of SKCM patients in TCGA was also obtained from TCGA Spliceseq website. The results of SNP analysis revealed that there were obvious mutations in both gene sets, and most of the changes in CNV were concentrated on the amplification of copy number. The variable splicing analysis showed that the Signature gene-A set mainly had ES in SKCM patients, while the Signature gene-B set had no obvious form of variable splicing. This result highlighted the influence of the Signature gene-A set on the immune landscape. According to the expression of Signature gene-A and -B sets, we screened out the more relevant lncRNAs as immune-related lncRNAs and constructed a ceRNA regulatory network of mRNA–miRNA–LncRNA interaction.

To create a better clinical prediction model for different SKCM patients, a novel prognostic-related risk scoring system was developed. First, we incorporated Signature gene-A and -B sets into the univariate Cox model and obtained 233 prognostic-related genes. A subsequent LASSO Cox analysis was performed for dimensionality reduction and model construction, and finally, 13 hub genes were obtained. This novel scoring system had good predictive ability for OS. The AUC of 1-year, 3-year, and 5-year OS was 0.713, 0.694, and 0.734, respectively. Furthermore, GSEA was employed to evaluate the correlation between the high- and low-risk groups and biological characteristics. Pathways, such as metabolism-related pathways and oxidative phosphorylation, were mainly enriched in the high-risk group, while immune response-related pathways, including cytokine signaling pathway, JAK–STAT signaling pathway, and natural killer cell-mediated cytotoxicity, were significantly enriched in low-risk patients. This finding is consistent with a previous report (Hu et al., 2020). Moreover, the expression of TME cells, as well as some other pathways, such as angiogenesis, mismatch-related characteristics, and matrix-related characteristics, was significantly different between the high- and low-risk groups. Finally, we assessed the correlation between the risk score and the clinical classification and pathological characteristics of SKCM patients and found that low-risk scores were often associated with patients’ TME cluster-A classification and metastatic SKCM. In different tumors, 13 hub genes showed different prognostic effects. Furthermore, the results of univariate and multivariate Cox analyses showed that risk scores could be identified as independent risk factors for predicting the prognosis of patients with SKCM. We subsequently selected statistically significant clinical indicators in the multivariate Cox model to construct a nomogram to predict the OS of patients with SKCM. The C-index indicates that the model had a high degree of discrimination. Validation of the calibration curve also revealed good concordance between the estimated values and the actual probability. To our knowledge, this is the first study to explore factors that directly alter the TME in SKCM. Thus, our immune model is more predictable for facilitating treatment than TMB and other predictive factors.

Among the six prognostic signatures, Ubiquitin/ISG15-conjugating enzyme E2 L6 (Ube2L6) has been reported to promote insulin resistance and hepatic steatosis and is related to cisplatin resistance (Murakami et al., 2020; Wei et al., 2021). SRPX2 is invasive by upregulating the FAK/SRC/ERK pathway and can lead to pancreatic cancer drug resistance in PI3K/AKT in lung cancer. Additionally, the NFATc3/SRPX2 axis participates in human embryonic stem cell differentiation, indicating that SRPX2 plays a critical role in pan-cancers (Gao et al., 2020; Li et al., 2020; Chen et al., 2021). SRPX2 is a component of the extracellular matrix, which is important for regulating tumor formation, as demonstrated in diverse tumors, such as colorectal cancer (Øster et al., 2013), gastrointestinal cancer (Tanaka et al., 2012), prostate cancer (Zhang et al., 2018), and pancreatic cancer (Gao et al., 2015). Interferon-induced protein with tetratricopeptide repeats protein may be a new therapeutic target for cancer therapy and can be used as a prognostic marker for cancers, such as glioblastoma and pancreatic cancer (Pidugu et al., 2019). In previous studies, CLEC4E was found to be related to tuberculosis and could be used to constrain tuberculosis through autophagy against drug-resistant strains (Kabuye et al., 2019; Pahari et al., 2020). Killer cell immunoglobulin-like receptor 2DL4 (KIR2DL4) is expressed by NK cells. According to some reports, KIR2DL4 may be an intervention for cancer immunotherapy (Attia et al., 2020). In our IHC analysis, some of these relationships were verified in SKCM patients. Only few studies have examined the relationship between SKCM and these hub genes. Thus, our study revealed their underlying relationships and found a novel important signature.

Therefore, the novel six-gene signature, which was developed to evaluate the comprehensiveness of TME in SKCM, is a reliably developed gene classifier that can be used to predict prognosis and guide more accurate molecular therapy.

## Conclusion

In conclusion, we have developed and verified an unprecedented set of effective prognostic markers based on immune infiltrating cells, which have certain potential application value in predicting the clinical prognosis of patients with SKCM and the benefit of immunotherapy. This study provides a systematic view of the immune-related characteristics in SKCM and suggests their good prognostic performance.

## Data Availability Statement

The datasets presented in this study can be found in online repositories. The names of the repository/repositories and accession number(s) can be found in the article/Supplementary Material.

## Ethics Statement

The studies involving human participants were reviewed and approved by The First People’s Hospital of Foshan Subject Review Board. The patients/participants provided their written informed consent to participate in this study. Written informed consent was obtained from the individual(s) for the publication of any potentially identifiable images or data included in this manuscript.

## Author Contributions

SZ and YS conceived and designed the study and wrote the manuscript. TC, JW, JH, JL, and YS contributed to the data acquisition. RY and XC analyzed the data. All authors read and approved the final manuscript.

## Conflict of Interest

The authors declare that the research was conducted in the absence of any commercial or financial relationships that could be construed as a potential conflict of interest.

## Publisher’s Note

All claims expressed in this article are solely those of the authors and do not necessarily represent those of their affiliated organizations, or those of the publisher, the editors and the reviewers. Any product that may be evaluated in this article, or claim that may be made by its manufacturer, is not guaranteed or endorsed by the publisher.

## Footnotes

1
http://www.ncbi.nlm.nih.gov/geo

2
http://gepia.cancer-pku.cn/index.html

3
http://software.broadinstitute.org/gsea/index.jsp

4
http://starbase.sysu.edu.cn/

## Supplementary Material

Supplementary Figure 1 The correlation between the expression of different immune cells in TCGA-SKCM cohorts.

## Abbreviations

TME: tumor microenvironment
AUC: area under the curve
TMB: tumor mutation burden
SKCM: skin cutaneous melanoma
ICI: immune checkpoint inhibitors
GEO: gene expression omnibus
TCGA: the cancer genome atlas
GSVA: gene set variation analysis
GSEA: gene set enrichment analysis
OS: overall survival
PCA: principal component analysis.

## References

[B1] Attia J. V. D. Dessens C. E. van de Water R. Houvast R. D. Kuppen P. J. K. Krijgsman D. (2020). The molecular and functional characteristics of HLA-G and the interaction with its receptors: where to intervene for cancer immunotherapy? *Int. J. Mol. Sci.* 21:8678. 10.3390/ijms21228678 33213057 PMC7698525

[B2] Chen H. Boutros P. C. (2011). VennDiagram: a package for the generation of highly-customizable Venn and Euler diagrams in R. *BMC Bioinformatics* 12:35. 10.1186/1471-2105-12-35 21269502 PMC3041657

[B3] Chen H. Zeng Y. Shao M. Zhao H. Fang Z. Gu J. (2021). Calcineurin A gamma and NFATc3/SRPX2 axis contribute to human embryonic stem cell differentiation. *J. Cell Physiol.* 236 5698–5714. 10.1002/jcp.30255 33393109

[B4] Dreno B. Thompson J. F. Smithers B. M. Santinami M. Jouary T. Gutzmer R. (2018). MAGE-A3 immunotherapeutic as adjuvant therapy for patients with resected, MAGE-A3-positive, stage III melanoma (DERMA): a double-blind, randomised, placebo-controlled, phase 3 trial. *Lancet Oncol.* 19 916–929. 10.1016/s1470-2045(18)30254-7 29908991

[B5] Fecher L. A. Cummings S. D. Keefe M. J. Alani R. M. (2007). Toward a molecular classification of melanoma. *J. Clin. Oncol.* 25 1606–1620. 10.1200/jco.2006.06.0442 17443002

[B6] Gao Z. Wu J. Wu X. Zheng J. Ou Y. (2020). SRPX2 boosts pancreatic cancer chemoresistance by activating PI3K/AKT axis. *Open Med. (Wars.)* 15 1072–1082. 10.1515/med-2020-0157 33336063 PMC7718643

[B7] Gao Z. Zhang J. Bi M. Han X. Han Z. Wang H. (2015). SRPX2 promotes cell migration and invasion via FAK dependent pathway in pancreatic cancer. *Int. J. Clin. Exp. Pathol.* 8 4791–4798. 26191169 PMC4503041

[B8] Goodson A. G. Grossman D. (2009). Strategies for early melanoma detection: approaches to the patient with nevi. *J. Am. Acad. Dermatol.* 60 719–735. 10.1016/j.jaad.2008.10.065 19389517 PMC2690513

[B9] Grossman R. L. Heath A. P. Ferretti V. Varmus H. E. Lowy D. R. Kibbe W. A. (2016). Toward a shared vision for cancer genomic data. *N. Engl. J. Med.* 375 1109–1112. 10.1056/NEJMp1607591 27653561 PMC6309165

[B10] Hanahan D. Weinberg R. A. (2011). Hallmarks of cancer: the next generation. *Cell* 144 646–674. 10.1016/j.cell.2011.02.013 21376230

[B11] He C. Zhang Y. Jiang H. Niu X. Qi R. Gao X. (2020). Identification of differentially expressed methylated genes in melanoma versus nevi using bioinformatics methods. *PeerJ* 8:e9273. 10.7717/peerj.9273 32547879 PMC7275674

[B12] Hodi F. S. O’Day S. J. McDermott D. F. Weber R. W. Sosman J. A. Haanen J. B. (2010). Improved survival with ipilimumab in patients with metastatic melanoma. *N. Engl. J. Med.* 363 711–723. 10.1056/NEJMoa1003466 20525992 PMC3549297

[B13] Hu B. Wei Q. Li X. Ju M. Wang L. Zhou C. (2020). Development of an IFNγ response-related signature for predicting the survival of cutaneous melanoma. *Cancer Med.* 9 8186–8201. 10.1002/cam4.3438 32902917 PMC7643661

[B14] Hu X. Yuan L. Ma T. (2020). Mechanisms of JAK-STAT signaling pathway mediated by CXCL8 gene silencing on epithelial-mesenchymal transition of human cutaneous melanoma cells. *Oncol. Lett.* 20 1973–1981. 10.3892/ol.2020.11706 32724443 PMC7377181

[B15] Jameson-Lee M. Luke J. J. (2021). Ipilimumab combination dosing: less is more. *Clin Cancer Res.* 10.1158/1078-0432.Ccr-21-2406 34341015 PMC8807780

[B16] Kabuye D. Chu Y. Lao W. Jin G. Kang H. (2019). Association between CLEC4E gene polymorphism of mincle and pulmonary tuberculosis infection in a northern Chinese population. *Gene* 710 24–29. 10.1016/j.gene.2019.05.011 31075410

[B17] Koikawa K. Kibe S. Suizu F. Sekino N. Kim N. Manz T. D. (2021). Targeting Pin1 renders pancreatic cancer eradicable by synergizing with immunochemotherapy. *Cell* 10.1016/j.cell.2021.07.020 [Epub ahead of print] 34388391 PMC8557351

[B18] Leek J. T. Johnson W. E. Parker H. S. Jaffe A. E. Storey J. D. (2012). The sva package for removing batch effects and other unwanted variation in high-throughput experiments. *Bioinformatics* 28 882–883. 10.1093/bioinformatics/bts034 22257669 PMC3307112

[B19] Li X. Liu J. Sun H. Zou Y. Chen J. Chen Y. (2020). SRPX2 promotes cell proliferation and invasion via activating FAK/SRC/ERK pathway in non-small cell lung cancer. *Acta Biochim. Pol.* 67 165–172. 10.18388/abp.2020_5158 32550700

[B20] Marron T. U. Ryan A. E. Reddy S. M. Kaczanowska S. Younis R. H. Thakkar D. (2021). Considerations for treatment duration in responders to immune checkpoint inhibitors. *J. Immunother. Cancer* 9:e001901. 10.1136/jitc-2020-001901 33653801 PMC7929825

[B21] Mayakonda A. Lin D. C. Assenov Y. Plass C. Koeffler H. P. (2018). Maftools: efficient and comprehensive analysis of somatic variants in cancer. *Genome Res.* 28 1747–1756. 10.1101/gr.239244.118 30341162 PMC6211645

[B22] Mitchell T. C. Hamid O. Smith D. C. Bauer T. M. Wasser J. S. Olszanski A. J. (2018). Epacadostat plus pembrolizumab in patients with advanced solid tumors: phase I results from a multicenter, open-label phase I/II trial (ECHO-202/KEYNOTE-037). *J. Clin. Oncol.* 36 3223–3230. 10.1200/jco.2018.78.9602 30265610 PMC6225502

[B23] Murakami M. Izumi H. Kurita T. Koi C. Morimoto Y. Yoshino K. (2020). UBE2L6 is involved in cisplatin resistance by regulating the transcription of ABCB6. *Anticancer Agents Med. Chem.* 20 1487–1496. 10.2174/1871520620666200424130934 32329696

[B24] Newman A. M. Liu C. L. Green M. R. Gentles A. J. Feng W. Xu Y. (2015). Robust enumeration of cell subsets from tissue expression profiles. *Nat. Methods* 12 453–457. 10.1038/nmeth.3337 25822800 PMC4739640

[B25] Øster B. Linnet L. Christensen L. L. Thorsen K. Ongen H. Dermitzakis E. T. (2013). Non-CpG island promoter hypomethylation and miR-149 regulate the expression of SRPX2 in colorectal cancer. *Int. J. Cancer* 132 2303–2315. 10.1002/ijc.27921 23115050

[B26] Pahari S. Negi S. Aqdas M. Arnett E. Schlesinger L. S. Agrewala J. N. (2020). Induction of autophagy through CLEC4E in combination with TLR4: an innovative strategy to restrict the survival of Mycobacterium tuberculosis. *Autophagy* 16 1021–1043. 10.1080/15548627.2019.1658436 31462144 PMC7469444

[B27] Pidugu V. K. Pidugu H. B. Wu M. M. Liu C. J. Lee T. C. (2019). Emerging functions of human IFIT proteins in cancer. *Front. Mol. Biosci.* 6:148. 10.3389/fmolb.2019.00148 31921891 PMC6930875

[B28] Quezada S. A. Peggs K. S. Simpson T. R. Allison J. P. (2011). Shifting the equilibrium in cancer immunoediting: from tumor tolerance to eradication. *Immunol. Rev.* 241 104–118. 10.1111/j.1600-065X.2011.01007.x 21488893 PMC3727276

[B29] Raskin L. Fullen D. R. Giordano T. J. Thomas D. G. Frohm M. L. Cha K. B. (2013). Transcriptome profiling identifies HMGA2 as a biomarker of melanoma progression and prognosis. *J. Invest. Dermatol.* 133 2585–2592. 10.1038/jid.2013.197 23633021 PMC4267221

[B30] Ritchie M. E. Phipson B. Wu D. Hu Y. Law C. W. Shi W. (2015). limma powers differential expression analyses for RNA-sequencing and microarray studies. *Nucleic Acids Res.* 43 e47. 10.1093/nar/gkv007 25605792 PMC4402510

[B31] Robin X. Turck N. Hainard A. Tiberti N. Lisacek F. Sanchez J. C. (2011). pROC: an open-source package for R and S+ to analyze and compare ROC curves. *BMC Bioinformatics* 12:77. 10.1186/1471-2105-12-77 21414208 PMC3068975

[B32] Siegel R. L. Miller K. D. Fuchs H. E. Jemal A. (2021). Cancer Statistics, 2021. *CA Cancer J. Clin.* 71 7–33. 10.3322/caac.21654 33433946

[B33] Subramanian A. Tamayo P. Mootha V. K. Mukherjee S. Ebert B. L. Gillette M. A. (2005). Gene set enrichment analysis: a knowledge-based approach for interpreting genome-wide expression profiles. *Proc. Natl. Acad. Sci. U. S. A.* 102 15545–15550. 10.1073/pnas.0506580102 16199517 PMC1239896

[B34] Tanaka K. Arao T. Tamura D. Aomatsu K. Furuta K. Matsumoto K. (2012). SRPX2 is a novel chondroitin sulfate proteoglycan that is overexpressed in gastrointestinal cancer. *PLoS One* 7:e27922. 10.1371/journal.pone.0027922 22242148 PMC3252299

[B35] Tang Z. Kang B. Li C. Chen T. Zhang Z. (2019). GEPIA2: an enhanced web server for large-scale expression profiling and interactive analysis. *Nucleic Acids Res.* 47 W556–W560. 10.1093/nar/gkz430 31114875 PMC6602440

[B36] Thompson J. F. Scolyer R. A. Kefford R. F. (2009). Cutaneous melanoma in the era of molecular profiling. *Lancet* 374 362–365. 10.1016/s0140-6736(09)61397-0 19647595

[B37] Tomczak K. Czerwińska P. Wiznerowicz M. (2015). The Cancer Genome Atlas (TCGA): an immeasurable source of knowledge. *Contemp. Oncol. (Pozn.)* 19 A68–A77. 10.5114/wo.2014.47136 25691825 PMC4322527

[B38] Topalian S. L. Hodi F. S. Brahmer J. R. Gettinger S. N. Smith D. C. McDermott D. F. (2012). Safety, activity, and immune correlates of anti-PD-1 antibody in cancer. *N. Engl. J. Med.* 366 2443–2454. 10.1056/NEJMoa1200690 22658127 PMC3544539

[B39] Wagner N. B. Weide B. Gries M. Reith M. Tarnanidis K. Schuermans V. (2019). Tumor microenvironment-derived S100A8/A9 is a novel prognostic biomarker for advanced melanoma patients and during immunotherapy with anti-PD-1 antibodies. *J. Immunother. Cancer* 7:343. 10.1186/s40425-019-0828-1 31806053 PMC6896585

[B40] Wei W. Li Y. Li Y. Li D. (2021). Adipose-specific knockout of ubiquitin-conjugating enzyme E2L6 (Ube2l6) reduces diet-induced obesity, insulin resistance, and hepatic steatosis. *J. Pharmacol. Sci.* 145 327–334. 10.1016/j.jphs.2020.12.008 33712284

[B41] Wilkerson M. D. Hayes D. N. (2010). ConsensusClusterPlus: a class discovery tool with confidence assessments and item tracking. *Bioinformatics* 26 1572–1573. 10.1093/bioinformatics/btq170 20427518 PMC2881355

[B42] Xu L. Shen S. S. Hoshida Y. Subramanian A. Ross K. Brunet J. P. (2008). Gene expression changes in an animal melanoma model correlate with aggressiveness of human melanoma metastases. *Mol. Cancer Res.* 6 760–769. 10.1158/1541-7786.Mcr-07-0344 18505921 PMC2756991

[B43] Yu G. Wang L. G. Han Y. He Q. Y. (2012). clusterProfiler: an R package for comparing biological themes among gene clusters. *OMICS* 16 284–287. 10.1089/omi.2011.0118 22455463 PMC3339379

[B44] Zeng L. Fan X. Wang X. Deng H. Zhang K. Zhang X. (2019). Bioinformatics analysis based on multiple databases identifies hub genes associated with hepatocellular carcinoma. *Curr. Genomics* 20 349–361. 10.2174/1389202920666191011092410 32476992 PMC7235396

[B45] Zhang H. Meltzer P. Davis S. (2013). RCircos: an R package for Circos 2D track plots. *BMC Bioinformatics* 14:244. 10.1186/1471-2105-14-244 23937229 PMC3765848

[B46] Zhang M. Li X. Fan Z. Zhao J. Liu S. Zhang M. (2018). High SRPX2 protein expression predicts unfavorable clinical outcome in patients with prostate cancer. *Onco. Targets Ther.* 11 3149–3157. 10.2147/ott.S158820 29881288 PMC5983007

[B47] Zhao X. Shen J. Ivaturi V. Gopalakrishnan M. Feng Y. Schmidt B. J. (2020). Model-based evaluation of the efficacy and safety of nivolumab once every 4 weeks across multiple tumor types. *Ann. Oncol.* 31 302–309. 10.1016/j.annonc.2019.10.015 31959348

