# Supplementary figures and images for: The Landscape of the Tumor Microenvironment in Skin Cutaneous Melanoma Reveals a Prognostic and Immunotherapeutically Relevant Gene Signature
Source: Front Cell Dev Biol. 2021 Oct 1;9:739594. doi: 10.3389/fcell.2021.739594 (PMC8517264; doi:10.3389/fcell.2021.739594)

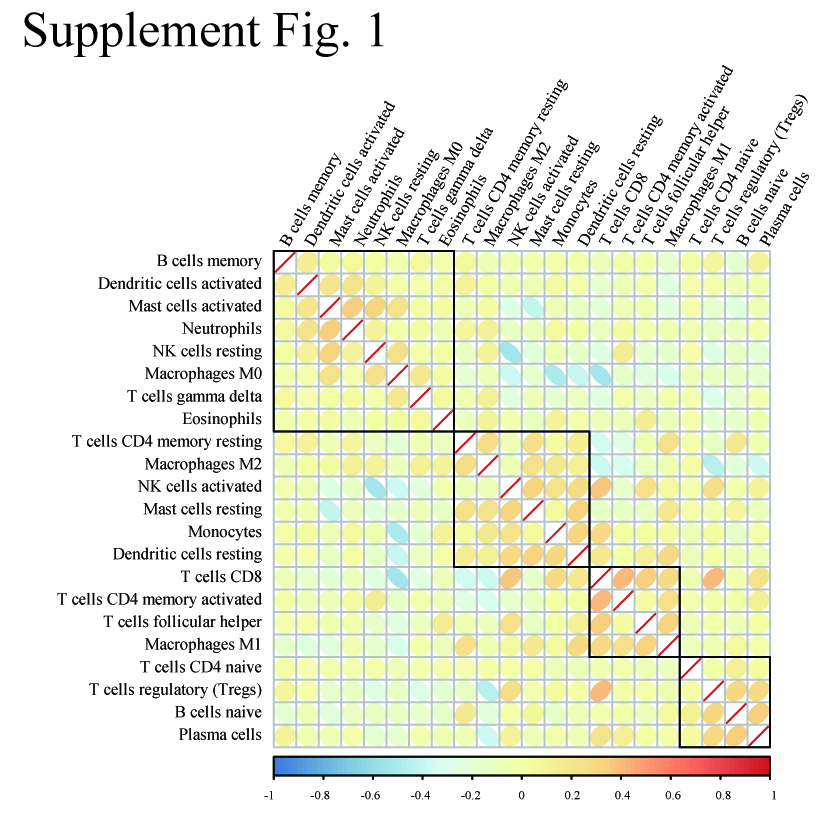

Supplement: Supplementary Figure 1 — The correlation between the expression of different immune cells in TCGA-SKCM cohorts. [file Image_1.TIF]
